# Efgartigimod in Very-Late-Onset Generalized Myasthenia Gravis: A Real-World Study on Effectiveness and Safety

**DOI:** 10.2174/011570159X415163251205123550

**Published:** 2026-01-13

**Authors:** Chi Ma, Jingyi Shen, Ying Zhu, Benqiao Wang, Ruixia Zhu

**Affiliations:** 1 Department of Neurology, The First Affiliated Hospital of China Medical University, Key Laboratory of Neurological Disease Big Data of Liaoning Province, Shenyang Clinical Medical Research Center for Difficult and Serious Diseases of the Nervous System, Shenyang, China

**Keywords:** Efgartigimod, AChR-gMG, very-late-onset, effectiveness, safety, corticosteroid reduction

## Abstract

**Introduction:**

This study evaluated the safety and effectiveness of efgartigimod in patients with Very-Late-Onset Generalized Myasthenia Gravis (VLOGMG), a population that often presents with more severe symptoms and limited treatment responses.

**Methods:**

Forty-two patients aged ≥ 65 years were retrospectively included. Clinical assessments, including MG activities of Daily Living (MG-ADL), Quantitative Myasthenia Gravis (QMG) scores, prednisone dosage, laboratory data, and adverse events, were recorded at each follow-up.

**Results:**

At week 4, 97.6% (41/42; 95% CI, 87.4-99.9) of patients were MG-ADL responders (≥ 2-point improvement). Notably, 83.3% (95% CI, 69.4-92.8) maintained response through week 12 (*p* < 0.001). Clinically meaningful improvement (CMI, ≥3-point QMG decrease) occurred in 97.6% of patients (41/42; 95% CI, 87.4-99.9), with a mean response time of 6.37 ± 5.46 days (95% CI, 4.63-8.11). Minimal symptom expression (MSE, MG-ADL score of 0 or 1) was achieved in 45% (95% CI, 30-61%), 60% (95% CI, 44-74%), and 45% (95% CI, 30-61%) of patients at weeks 4, 8, and 12, respectively. Prednisone dosage was tapered from a median of 20 (20, 30) mg/day to 10 (5, 15) mg/day by week 12. Most patients (88.1%) had ≥ 1 comorbidity, and 61.9% had multimorbidity. Efgartigimod was well tolerated, without evidence of worsening of pre-existing conditions.

**Discussion:**

Efgartigimod provided rapid symptom relief in older adults with VLOGMG and demonstrated steroid-sparing benefits across patients with various comorbidities. The high response rates and sustained improvement suggest that early use of fast-acting therapies may serve as a bridge to conventional long-term treatments. Larger prospective studies are warranted to confirm these findings.

**Conclusion:**

Efgartigimod is associated with clinical benefit in patients with VLOGMG, allowing corticosteroid reduction without compromising comorbidity stability. Early initiation may enable faster disease control and more durable responses.

## 1 INTRODUCTION

Myasthenia Gravis (MG) is an autoimmune disorder characterized by muscle weakness and fatigue, mediated by autoantibodies against the neuromuscular junction, primarily targeting Acetylcholine Receptor (AChR), Muscle-Specific Kinase (MuSK), or low-density Lipoprotein Receptor-related Protein 4 (LRP4) [1]. MG is highly heterogeneous, with subgroups defined by clinical features, autoantibody profiles, age at onset, and thymus pathology. AChR-MG exhibits a bimodal age-of-onset distribution, peaking at approximately 30 and 70-80 years [2]. Patients are commonly classified as early-onset (18-49 years), late-onset (50-64 years), or very-late-onset (≥ 65 years, VLOGMG) [3].

Several studies have been conducted to investigate the clinical profile of VLOGMG. Cortés-Vicente *et al*. analyzed 939 patients, of whom 424 (45.2%) had VLOGMG, predominantly those with AChR-antibody-positive. These patients were less likely to develop thymomas but had thymic atrophy compared with patients with early-onset MG. Additionally, the proportion of males was higher [4]. Recently, Tang *et al*. using a Chinese MG database—reported that VLOGMG patients (161 of 1,160) had a poorer prognosis than younger patients, with fewer achieving or maintaining Minimal Manifestation Status (MMS) and higher MG-related mortality [5]. Older patients often take multiple medications, which may exacerbate MG or interact with immunosuppressants, and they exhibit increased susceptibility to adverse reactions [4]. These observations underscore the need for individualized treatment strategies for VLOGMG, which may be informed by age-related neuromuscular vulnerabilities. FcRn inhibition may have particular implications in older adults with MG, possibly related to age-associated changes in IgG metabolism and immune regulation, which warrants further investigation.

Efgartigimod, a neonatal Fc receptor (FcRn) antagonist, promotes Immunoglobulin G (IgG) degradation and reduces serum antibody levels [6]. In the Phase III ADAPT trial, efgartigimod demonstrated rapid and significant symptom improvement; the mean ages of the efgartigimod and placebo groups were 45.9 and 48.2 years, respectively [7]. Despite growing real-world evidence supporting its effectiveness and safety, data on efgartigimod use in VLOGMG remain limited. To address this gap, we conducted a real-world study to evaluate the safety and effectiveness of this treatment in Chinese patients with VLOGMG.

## 2 METHOD

### 2.1 Study Design and Patient Selection

**Fig. (1):**
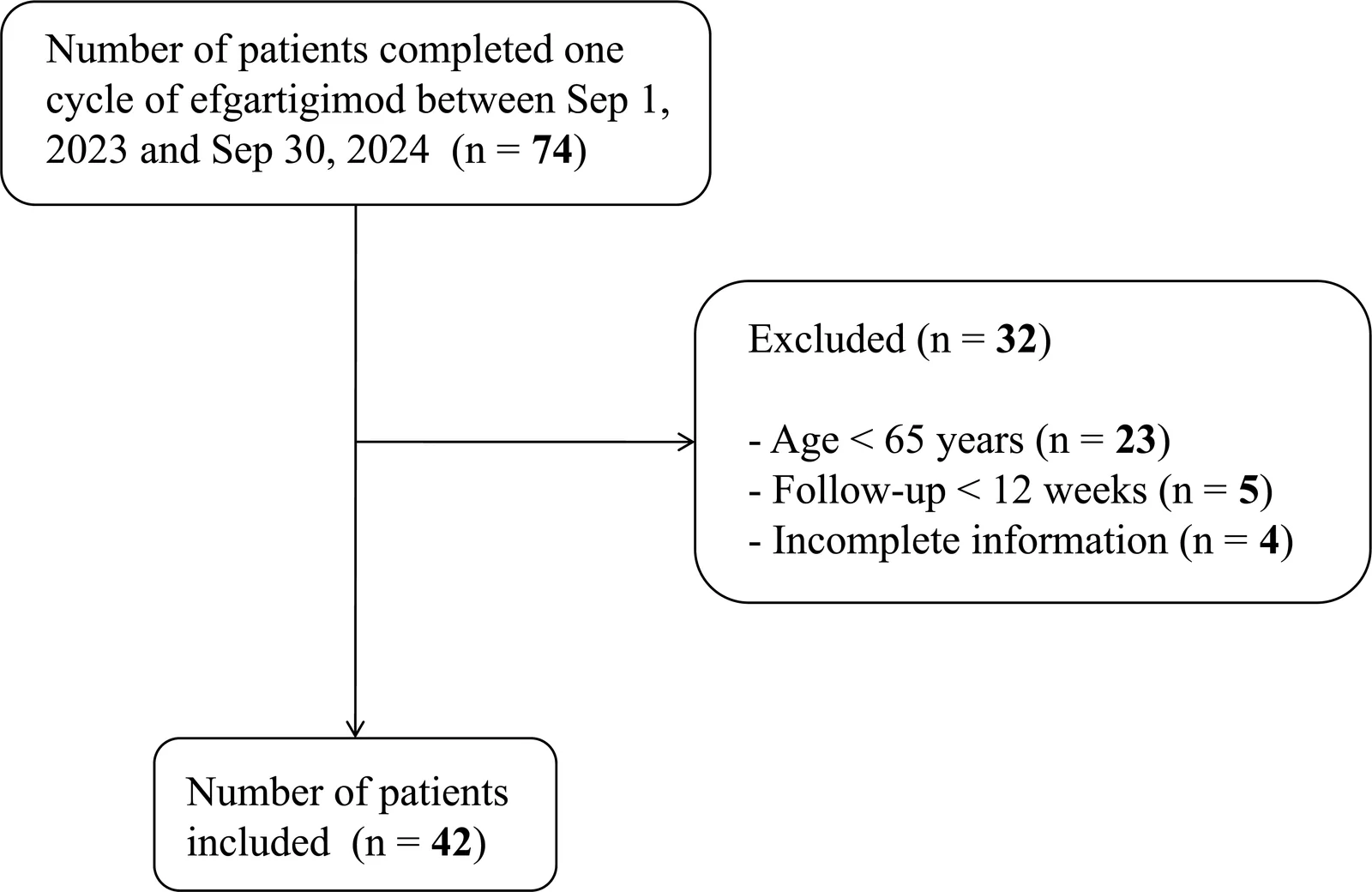
Flowchart of patient selection and exclusion.

Forty-two patients with VLOGMG who completed at least one cycle of efgartigimod treatment between September 1, 2023, and September 30, 2024, with a 12-week follow-up, were finally included in this single-center retrospective study (Fig. **1**). The inclusion criteria were as follows: (1) age ≥ 65 years; (2) a confirmed diagnosis of generalized MG (gMG); (3) Myasthenia Gravis Foundation of America (MGFA) classification of grade II, III or IV; and (4) completion of at least one treatment cycle of efgartigimod with a minimum follow-up of 12 weeks. VLOGMG was defined as disease onset at or after 65 years of age. Detailed baseline clinical characteristics were recorded for all enrolled patients, including sex, age at MG onset, disease duration, comorbidities, and concurrent pharmacological treatments. Each efgartigimod treatment cycle consisted of four weekly intravenous infusions at a dose of 10 mg/kg. The timing of subsequent treatment cycles was determined flexibly by the treating physician, based on clinical exacerbation of MG-ADL scores. The baseline prednisone dose ranged from 20 to 30 mg/day and did not exceed 60 mg/day. In some patients, tacrolimus was administered instead of corticosteroids. This study was approved by the Ethics Committee of the First Affiliated Hospital of China Medical University (Protocol No. 2024-507-2, China) and conducted in accordance with the Declaration of Helsinki. Written informed consent was obtained from all participants.

### 2.2 Clinical Assessment of MG

Comprehensive assessments of QMG and MG-ADL scores were performed and recorded at baseline, week 1, week 2, week 3, week 4, week 8, week 12, and at the final follow-up visit. An exacerbation was defined as an increase in the QMG score of > 4 points, whereas a Clinically Meaningful Improvement (CMI) was defined as a decrease of > 3 points in QMG. An MG-ADL responder was defined as a patient who demonstrated an improvement of ≥ 2 points in the MG-ADL score. Minimal Symptom Expression (MSE) was considered to be the absence of functional limitations due to MG, corresponding to an MG-ADL score of 0 or 1.

### 2.3 Laboratory Investigations and Safety Evaluation

Adverse Events (AEs) were closely monitored throughout the treatment cycle, including infection, fever, headache, dizziness, nausea, rash, diarrhea, allergy reactions, and hypertension. Furthermore, the nature and potential worsening of comorbidities were also carefully documented. Injection Site Reaction (ISR), such as erythema, swelling, pruritus and pain within one week of the administration, was also recorded. Patients' AChR antibody titers and serum immunoglobulin levels were measured at baseline and at week 4. Treatment-related metabolic effects were monitored *via* serum lipid and albumin levels.

### 2.4 Statistical Analyses

Descriptive statistics summarized the demographic and clinical characteristics. Data analyses were conducted using GraphPad Prism 8.0 and IBM SPSS version 25.0 (SPSS Inc., Chicago, IL, USA). Changes in MG-ADL and QMG scores over time were analyzed using repeated-measures ANOVA, followed by Bonferroni-adjusted pairwise comparisons, and laboratory parameters were compared using paired t-tests, while χ^2^ tests were used to analyze multiple independent samples. Kaplan-Meier survival analysis was used to estimate time to MSE, and corresponding curves were plotted. Statistical significance was defined as *p* < 0.05.

## 3 RESULTS

### 3.1 Baseline Clinical Characteristics

The study included 42 patients with VLOGMG (13 males and 29 females) (Table **1**). The mean age at onset was 70.04 ± 3.68 years, and all patients were aged ≥ 65 years. Among them, 40 patients (95.2%) had AChR antibody-positive gMG, and 2 patients (4.8%) were triple-seronegative. According to the MGFA classification, 14.2% were classified as class II, 81.0% as class III, and 4.8% as class IV. Two patients (4.8%) had thymoma, both of whom had undergone thymectomy. Of the cohort, 12 patients were treated with tacrolimus (1 in combination with prednisone), while 26 received prednisone alone. Overall, 88.1% of patients had at least one comorbidity, and 61.9% had multimorbidity. The most common comorbidities included diabetes mellitus, hypertension, cerebrovascular disease, coronary heart disease, malignancy, and thyroid disorders.

### 3.2 Clinical Association and Steroid-Sparing Effect

**Fig. (2):**
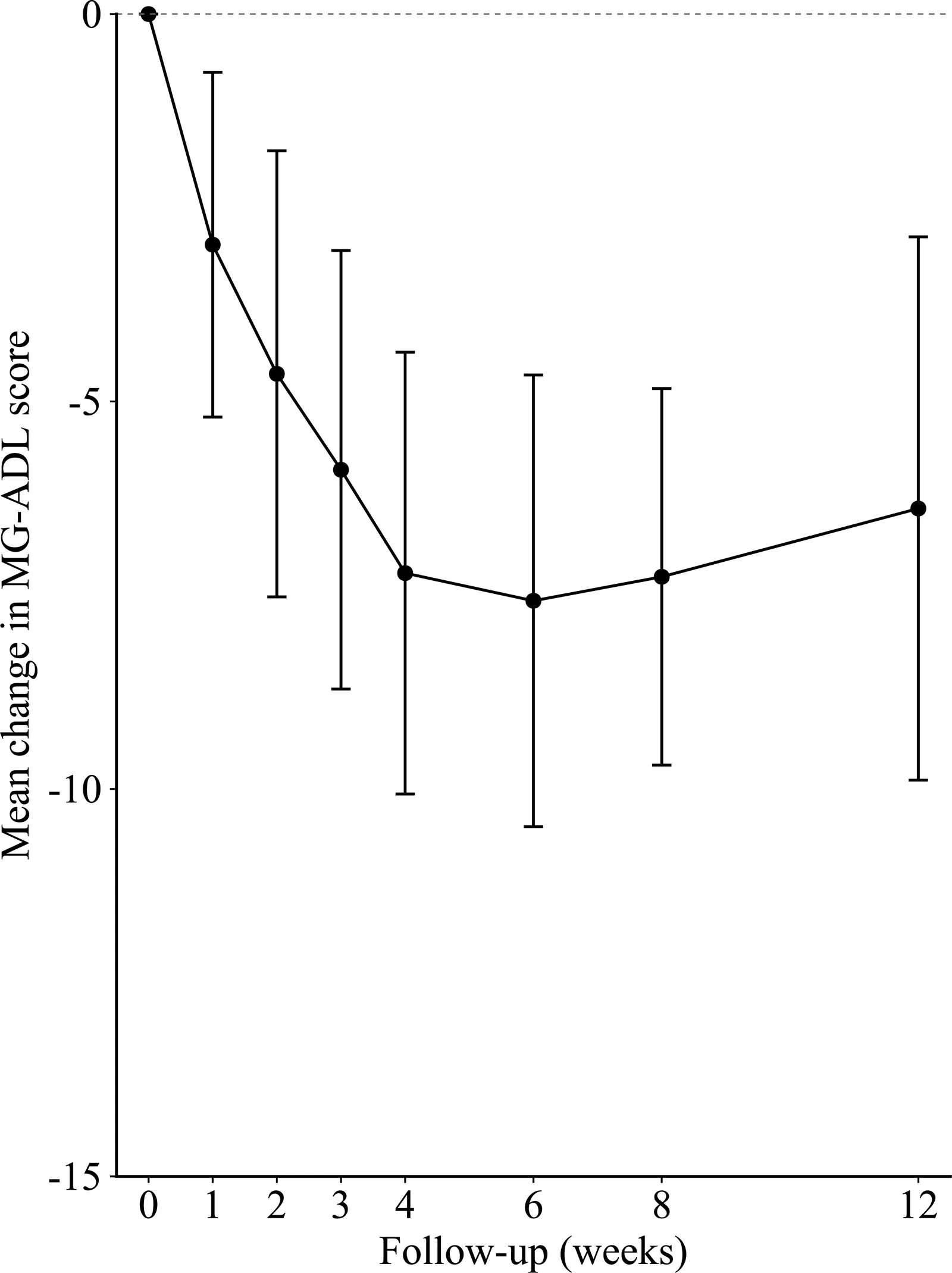
Mean change in MG-ADL scores from baseline to weeks 4, 8, and 12 (Mean ± SE) (*p* < 0.001). *MG-ADL* MG activities of Daily Living, *SE* Standard Error.

**Fig. (3):**
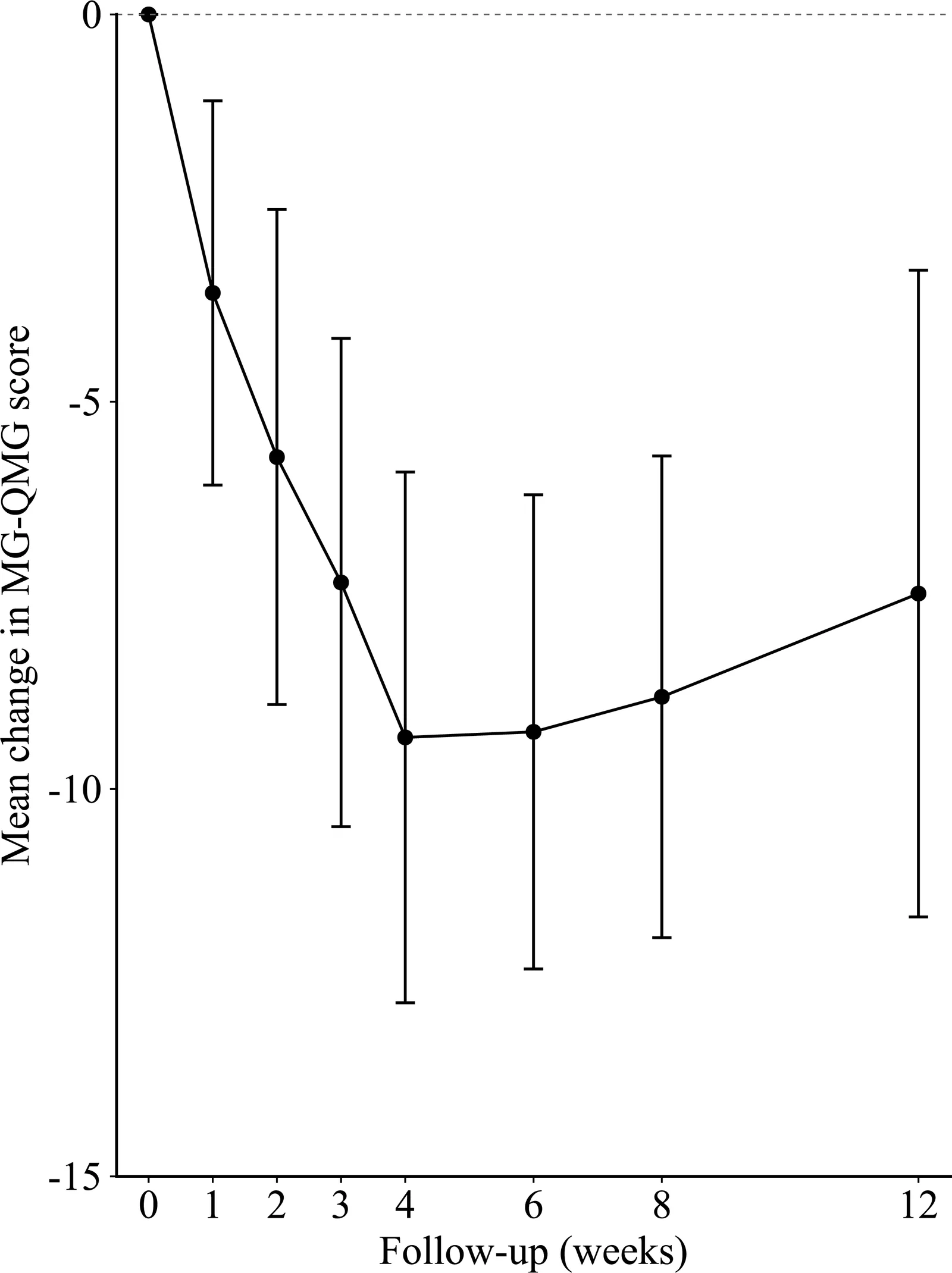
Mean change in QMG scores from baseline to weeks 4, 8, and 12 (*p* < 0.001). *QMG* Quantitative Myasthenia Gravis, *SE* Standard Error.

**Fig. (4):**
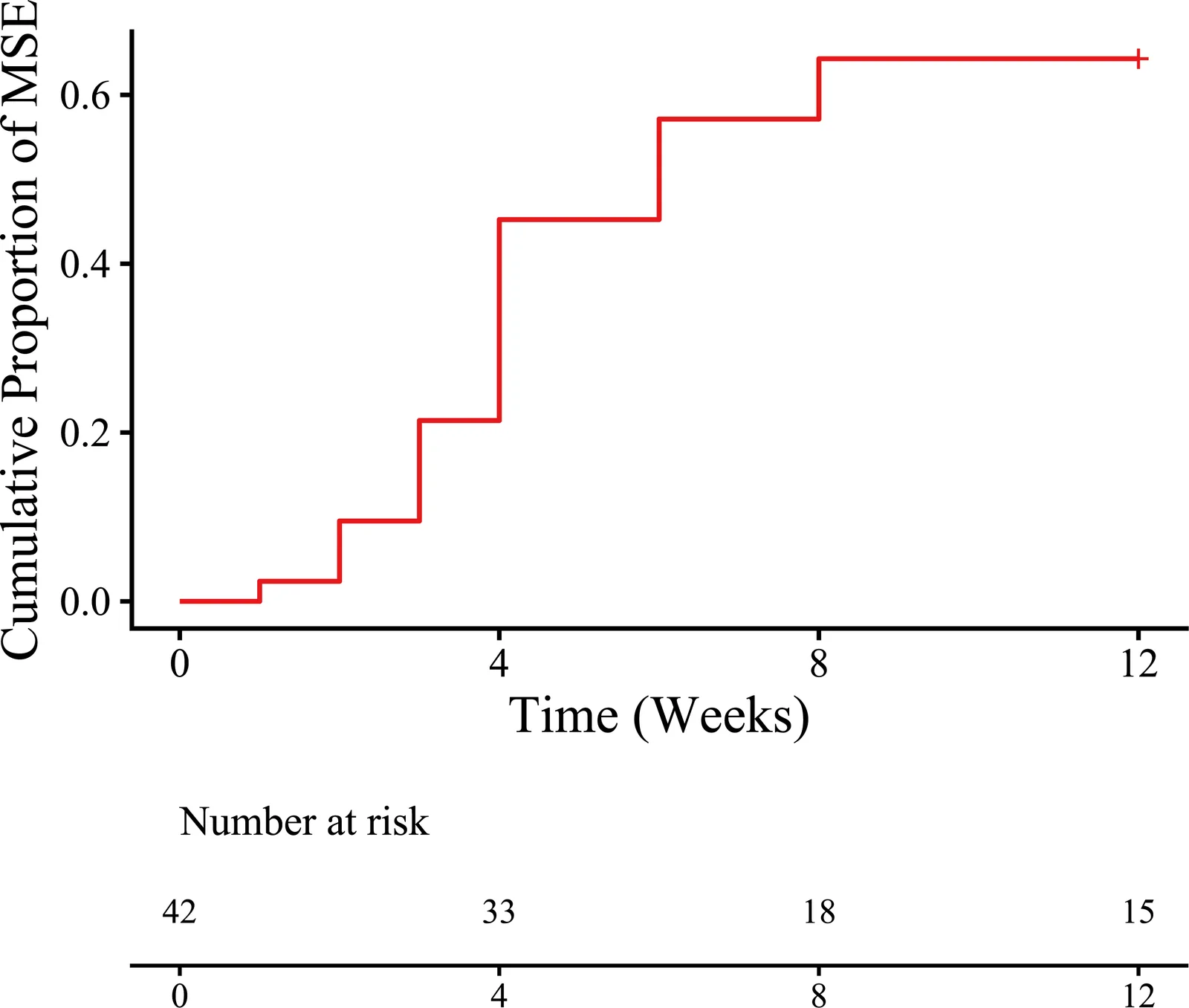
Kaplan-Meier curve of the cumulative proportion of patients achieving MSE over the 12-week follow-up. *MSE* Minimal symptom expression.

**Fig. (5):**
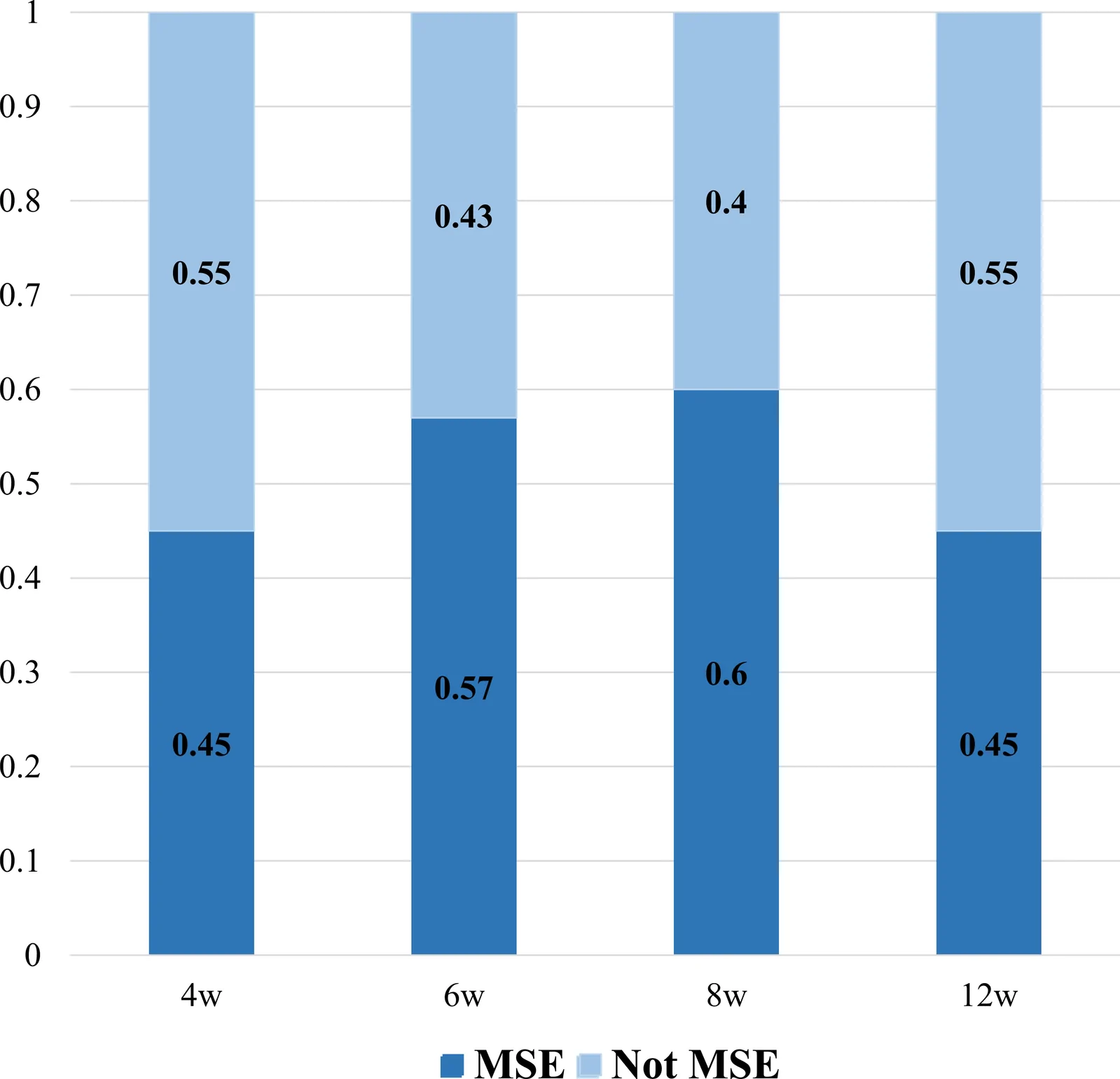
Distribution of patients who did or did not achieve MSE at weeks 4, 6, 8, and 12.

**Fig. (6):**
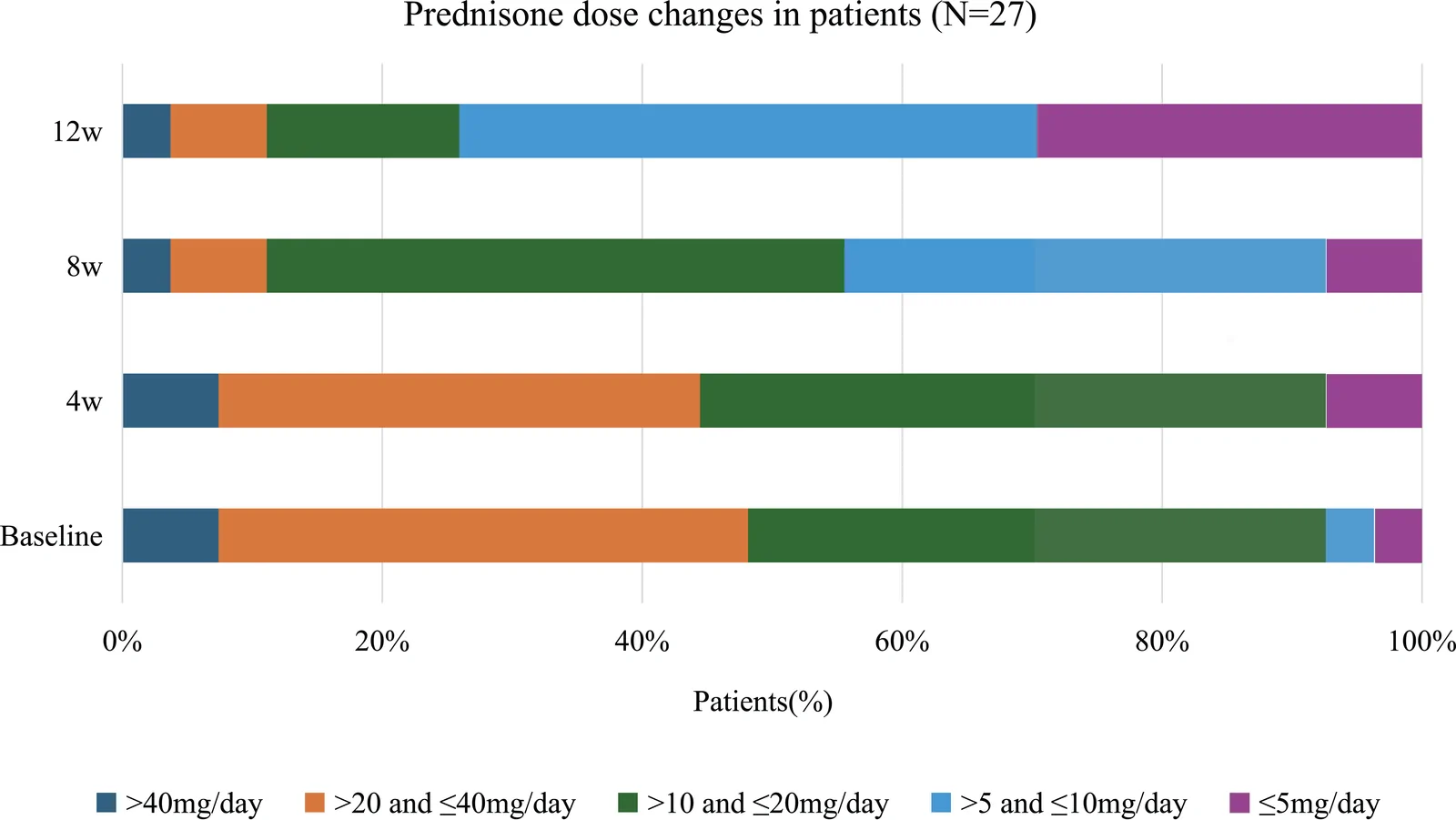
Proportion of patients (n = 27) receiving different prednisone dose ranges (mg/day) at baseline and at weeks 4, 8, and 12.

Among all participants, 97.6% (95% CI, 87.4-99.9) were MG-ADL responders. Mean MG-ADL scores decreased by 7.21 ± 2.85, 7.26 ± 2.43, and 6.38 ± 3.50 points at weeks 4, 8, and 12, respectively (*p* < 0.001, SE: 0.440, 0.375, 0.540) (Fig. **2**), with 83.3% (95% CI, 69.4-92.8) of responders maintaining improvement through the 12-week follow-up. Similarly, mean QMG scores declined from 13.00 ± 4.24 at baseline to 9.33 ± 3.42, 8.81 ± 3.11, and 7.47 ± 4.17 points at weeks 4, 8, and 12, respectively (*p* < 0.001, SE: 0.654, 0.528, 0.480, 0.643) (Fig. **3**). MSE was achieved by 45% (95% CI, 30-61%), 60% (95% CI, 44-74%), and 45% (95% CI, 30-61%) of patients at weeks 4, 8, and 12, respectively (Figs. **4** and **5**). Overall, 97.6% of patients attained CMI, with a mean response duration of 6.37 ± 5.46 days (95% CI, 4.63-8.11), whereas only one patient remained unresponsive due to fractures and malnutrition. Notably, four patients (9.5%) received a second cycle of efgartigimod: two had malignancies with thymic abnormalities, and two declined conventional immunosuppressants due to adverse effects and were maintained on repeated efgartigimod monotherapy for symptom control. Among patients receiving prednisone throughout efgartigimod treatment (n = 27), the median daily dose decreased from 20 (20, 30) mg/day at baseline to 10 (5, 15) mg/day at week 12 (Fig. **6**), with a median total prednisone dose over 12 weeks of 1470 (1050, 1890) mg. All patients were able to taper their steroid dose to ≤ 10 mg/day by 6 months, demonstrating a steroid-sparing effect, and no steroid-related adverse events (*e.g*., osteoporosis, fractures, hyperglycemia, or hypertension) were observed.

### 3.3 IgG and AchR Antibody Levels

The mean IgG level decreased from 10.63 (9.89, 13.38) g/L at baseline to 4.31 (3.42, 5.34) g/L at week 4. The IgG nadir, defined as the week-4 value due to the available measurements, was 4.31 (3.42, 5.34) g/L, suggesting a possible risk of hypogammaglobulinemia. Although none of these cases were associated with severe infections, the clinical implications of hypogammaglobulinemia warrant further attention. Immunoglobulin A (IgA), Immunoglobulin M (IgM) and albumin levels exhibited irregular changes between baseline and week 4. The median AChR antibody titer declined from 30.14 (9.87, 54.75) nmol/L to 5.5 (2.12, 15.1) nmol/L by the end of one treatment cycle. Among the monitored B-cell parameters, a decrease in CD19+ cells was observed in four patients, consistent with previous reports that efgartigimod reduces the proportion of memory B cells [8]. Further studies are required to elucidate how efgartigimod, which facilitates antibody clearance, influences adaptive immunity, given the central role of B cells and plasma cells in the upstream pathogenesis of MG *via* production of pathogenic autoantibodies.

### 3.4 Safety Profile

Efgartigimod was generally well tolerated in patients with VLOGMG, with a favorable safety profile (Table **2**). Adverse events were infrequent and mild, including muscle pain, headache, and upper respiratory tract infection in five patients, none of which affected MG symptoms or treatment. During follow-up, infections occurred in only one patient. No patients required hospitalization or emergency visits, and no worsening of comorbidities was observed.

## 4 DISCUSSION

Efgartigimod demonstrated a rapid symptom control in VLOGMG patients, and its early use may allow for lower corticosteroid doses, thereby reducing steroid-related adverse events and mitigating the worsening of comorbidities, which are more common in older adults. Therefore, it may serve as a therapeutic bridge, facilitating the transition to traditional immunosuppressive agents with a slower onset and providing better clinical outcomes.

Previous studies have indicated that VLOGMG patients are less likely to achieve MSE or maintain it for a prolonged period. Moreover, this population is characterized by a high prevalence of complications such as diabetes mellitus, cataracts, and osteoporosis, alongside an increased susceptibility to adverse drug reactions and delayed recovery when treated with conventional regimens [4, 9]. Bi *et al*. demonstrated that older age at onset is a critical risk factor for MG exacerbations, which often require hospitalization and escalation of appropriate therapy [10]. A retrospective study conducted in Finland in 2024 concluded that late-onset MG is associated with an elevated risk of requiring intensive care treatment, suggesting the need for early intervention [11]. Patients with exacerbation often experience severe deterioration despite escalation of immunosuppressive therapy and increased corticosteroid doses [12]. Several studies have evaluated the effectiveness and safety of various MG treatment regimens in this population. In 2022, Zheng *et al*. reported that tacrolimus monotherapy may be a feasible option for both initial and maintenance treatment [13], while in 2023, Yang *et al*. found that corticosteroids combined with low-dose tocilizumab may provide benefit in a small cohort of five VLOGMG patients [14]. Nevertheless, there are no studies that have evaluated the effectiveness and safety of fast-acting therapies, such as FcRn antagonists, in this specific population.

Aging-related degeneration of the Neuromuscular Junction (NMJ) may contribute to the increased vulnerability observed in VLOGMG. Structural and functional studies have shown that aged NMJs undergo fragmentation, reduced AChR density, and impaired synaptic remodeling, resulting in decreased transmission efficiency [15, 16]. In MG, pathogenic autoantibodies further aggravate these defects through AChR internalization, complement activation, and disorganization of the postsynaptic membrane [17]. Thus, older patients experience a “double hit” of age-related degeneration and immune-mediated injury, leading to more severe weakness and delayed recovery. Given this dual burden, efgartigimod may provide unique advantages for older adults by rapidly lowering pathogenic IgG levels, alleviating NMJ stress, and improving neuromuscular transmission. The geriatric pharmacology of FcRn inhibitors warrants further investigation, and targeted pharmacokinetic studies in older MG populations are expected to elucidate the mechanisms.

Our study enrolled older patients with a mean age of 72.1 years, which is higher than that reported in the ADAPT trial (45.9 years in the efgartigimod group and 48.2 years in the placebo group), and included five patients over 80 years old. Among all participants, 97.6% were MG-ADL responders, and 83.3% maintained a sustained response throughout 12 weeks, which is higher than the 68% reported in the ADAPT study. At week 8, the proportion of patients maintaining MSE in ADAPT was 40%, compared with 60% in our study. In addition, 63.1% of patients in the efgartigimod group of ADAPT were QMG responders (defined as a ≥ 3-point improvement in QMG score for at least 4 consecutive weeks), in contrast to 92.9% in our cohort. In the ADAPT study, the median cycle interval (the time between the last injection of a previous cycle and the first injection of the next) was 7.3 weeks, while in ADAPT+, it was about 5.8 weeks [7, 18]. Although repeated cycles were often required to maintain efficacy in ADAPT, 45% of our patients sustained MSE for up to 12 weeks after a single cycle, suggesting a durable response in this real-world cohort. The mean IgG reduction in our study was 54%, slightly lower than the 57% observed in the ADAPT+ overall population and the 61.3% reported in the AChR antibody-positive group of ADAPT. The age gap between the ADAPT study and our real-world cohort is particularly noteworthy. While randomized controlled trials often impose strict age limitations, our findings provide evidence supporting the use of efgartigimod in older patients.

Our study also demonstrated that efgartigimod administration was associated with reduced corticosteroid requirements, which in turn decreased the risk of steroid-related adverse events, thereby enhancing treatment safety in older adults. In all patients in the study, oral prednisone dosage did not need to be increased to the maximum tolerated level, and the duration of high-dose corticosteroid treatment was shortened, achieving symptom control without dose escalation. These findings indicate a clear steroid-sparing effect, reducing systemic inflammation and minimizing steroid-related side effects.

In our cohort, 88.1% of patients had at least one comorbidity, including hypertension, diabetes mellitus, hyperlipidemia, cerebrovascular disease, autoimmune thyroid disorders, or cardiovascular disease. The high prevalence of multimorbidity may lead to overall functional impairment. Furthermore, some older patients may take medications such as statins or beta-blockers, which are contraindicated in MG, or may be more susceptible to infections and multisystemic symptoms due to comorbidities. These factors pose major challenges to developing a safe medication regimen [9]. Multimorbidity, therefore, represents a critical clinical challenge, exerting an even greater effect on quality of life and prognosis than MG symptoms themselves. Clinicians should carefully evaluate potential drug interactions and contraindications to optimize individualized treatment strategies for older adults.

The presence of multimorbidity makes efgartigimod an attractive therapeutic option. In our study, efgartigimod demonstrated a favorable safety profile, with no treatment-related severe adverse events. Mild adverse effects, including muscle pain, headache, and diarrhea, were transient and did not necessitate treatment discontinuation. Pre-existing comorbidities remained stable throughout the treatment course.

## 5 STUDY LIMITATIONS

This study is limited by its single-center, single-arm, retrospective design and modest sample size, which constrain both statistical power and generalizability. In real-world clinical practice, patient selection and treatment accessibility may differ from those in controlled trials, which should be considered when interpreting these findings. The absence of randomization, combined with heterogeneity in baseline treatments, such as corticosteroids or tacrolimus, may have introduced selection bias and unmeasured confounding. Moreover, the relatively short follow-up period precludes assessment of long-term outcomes. Collectively, these limitations underscore the need for larger, multicenter randomized trials with extended follow-up to more definitively evaluate the efficacy and safety of efgartigimod in VLOGMG patients.

## CONCLUSION

Efgartigimod was associated with improved clinical outcomes and was well-tolerated in patients with VLOGMG, allowing for a reduction in corticosteroid dosage while maintaining the stability of comorbidities. Our study provides real-world evidence for efgartigimod use in older patients. Early treatment escalation may achieve rapid disease control and a more sustained clinical response. Larger, randomized trials are warranted to further define the optimal treatment strategy for efgartigimod in this population.

## Figures and Tables

**Table 1:** Baseline features of patients enrolled, n=42.

**Clinical Variates**	**Mean ± SD (Range) or Percentage (%)**
Total Number	42
Sex (%)	-
Male	13 (31%)
Female	29 (69%)
Onset Age (years old)	70.04±3.68
Age (years old)	72.1±4.58
≥80 years	5 (11.9%)
≥75 years	10 (23.8%)
Mean Follow-up Time (months)	6.9±3.64
Antibody status, n (%)	-
AChR	40 (95.2%)
MuSK	0
LRP4	0
Negative	2 (4.8%)
Thymoma (%)	2 (4.8%)
MGFA classification at efgartigimod initiation	-
II	6 (14.3%)
III	34 (81%)
IV	2 (4.8%)
Mean baseline QMG score	13±4.24
Mean baseline MG-ADL score	9.88±3.48
Treatment at efgartigimod initiation (%)	-
Pyridostigmine	41 (97.6%)
Prednisone	27 (64.3%)
Tacrolimus	12 (28.6%)
Comorbidities	**-**
None	5 (11.9%)
At least one comorbidity	37 (88.1%)
Single comorbidity	11 (26.2%)
Multiple comorbidities	26 (61.9%)
Hypertension	23 (54.8%)
Diabetes mellitus	16 (38.1%)
Hyperlipidemia	7 (16.7%)
Autoimmune thyroid disease	9 (21.4%)
Cerebrovascular disease	6 (14.3%)
Cardiovascular disease	3 (7.1%)
Malignancy	3 (7.1%)
Other disorders (hepatic insufficiency, renal insufficiency, gastritis, *etc.*)	8 (19%)
Patients received 2 cycles of efgartigimod (%)	5 (11.9%)
The median time between cycles (weeks)	8 (8, 13)

**Abbreviations**: SD standard deviation. MGFA, Myasthenia Gravis Foundation of America. QMG, Quantitative Myasthenia Gravis. ADL, Myasthenia Gravis Activities of Daily Living.

**Table 2:** Adverse events observed in the study.

**Adverse Events**	**Percentage**
Any adverse event	5 (11.9%)
Any serious adverse event	0
Any adverse event leading to discontinuation of efgartigimod	0
Headache	2 (4.8%)
Muscle pain	1 (2.4%)
Upper respiratory tract infection	2 (4.8%)

## Data Availability

All the data and supporting material are available within the article.

## LIST OF ABBREVIATIONS

AChR: Acetylcholine Receptor
CD19: Cluster of Differentiation 19
CMI: Clinically Meaningful Improvement (Note: a decrease in the QMG score of more than 3 points)
FcRn: Neonatal Fc Receptor
IgA: Immunoglobulin A
IgG: Immunoglobulin G
IgM: Immunoglobulin M
ISR: Injection Site Reaction
LRP4: Low-density Lipoprotein Receptor-Related Protein 4
MSE: Minimal Symptom Expression (Note: MG-ADL score of 0 or 1)
MG: Myasthenia Gravis
MGFA: Myasthenia Gravis Foundation of America
MG-ADL: MG Activities of Daily Living
MMS: Minimal Manifestation Status
MuSK: Muscle-specific Receptor Tyrosine Kinase
NMJ: Neuromuscular Junction
QMG: Quantitative Myasthenia Gravis
SD: Standard Deviation
VLOGMG: Very-late-onset Generalized Myasthenia Gravis (Note: disease onset at or after 65 years of age)

## STANDARDS OF REPORTING

STROBE guidelines were followed.
